# A CADASIL-Like Case with a Novel Noncysteine Mutation of the *NOTCH3* Gene and Granular Deposits in the Renal Arterioles

**DOI:** 10.1155/2015/431461

**Published:** 2015-03-09

**Authors:** Kuniyuki Nakamura, Tetsuro Ago, Akihiro Tsuchimoto, Nozomi Noda, Asako Nakamura, Toshiharu Ninomiya, Takeshi Uchiumi, Kazuhiko Tsuruya, Masahiro Kamouchi, Hiroaki Ooboshi, Takanari Kitazono

**Affiliations:** ^1^Department of Medicine and Clinical Science, Graduate School of Medical Sciences, Kyushu University, Fukuoka 812-8582, Japan; ^2^Department of Internal Medicine, Fukuoka Dental College Medical and Dental Hospital, Fukuoka 814-0193, Japan; ^3^Department of Clinical Chemistry and Laboratory Medicine, Graduate School of Medical Sciences, Kyushu University, Fukuoka 812-8582, Japan

## Abstract

We herein report the finding of a 62-year-old male, who developed dysarthria and dysphagia, with cerebral autosomal dominant arteriopathy with subcortical infarcts and leukoencephalopathy- (CADASIL-) like cerebral lesions. He also suffered from slowly progressive renal failure with the findings of granular deposits similar to electron-dense granular osmiophilic material in the renal arterioles. We found a novel heterozygous missense mutation of the *NOTCH3* gene, c.4039G>C in exon 24, resulting in a p.Gly1347Arg substitution in its extracellular domain. The noncysteine substitution may underlie the pathogenesis of white matter lesions in the brain and of the chronic renal failure in the present case.

## 1. Introduction

Cerebral autosomal dominant arteriopathy with subcortical infarcts and leukoencephalopathy (CADASIL) is an autosomal dominant vascular disorder, characterized by migraines, recurrent strokes, pseudobulbar palsy, and subcortical dementia [1]. The histopathological hallmark of CADASIL is the accumulation of electron-dense granular osmiophilic material (GOM) in the basal lamina [2]. Magnetic resonance (MR) images (MRI) in CADASIL patients show extensive cerebral white matter lesions and subcortical infarcts.

In recent years, many molecular genetic defects have been identified as factors involved in the pathogenesis of cerebral infarction. Among them, the* NOTCH3* gene on the chromosome locus 19p13 is the only known gene responsible for CADASIL [1, 3]. The* NOTCH3* gene encodes a large transmembrane receptor. Seventy percent of* NOTCH3* gene missense mutations are present in exons 3 and 4, which gain or lose a cysteine residue, causing abnormal disulfide bond formation in the epidermal growth factor- (EGF-) like domain within the extracellular domain of the protein [4, 5].

We herein report a case presenting with CADASIL-like cerebral lesions and symptoms in whom a novel noncysteine* NOTCH3* gene mutation in exon 24 was found within the EGF-like repeat domain. This patient also showed chronic renal failure with granular deposits in the renal arterioles.

## 2. Case Presentation

A 62-year-old right-handed male with chronic renal failure due to chronic glomerulonephritis began to experience difficulty with speech and swallowing and was admitted to our hospital one month after the onset of these symptoms. At the age of 35, he had proteinuria and was diagnosed with nephrotic syndrome and kidney injury. He was diagnosed with possible membranoproliferative glomerulonephritis (MPGN) based on the results of a renal biopsy (see below). His systolic blood pressure was kept in control within 125–140 mmHg. The patient's medications included amlodipine, carvedilol, furosemide, doxazosin, and sodium hydrogen carbonate. He had no history of smoking or any other toxic habits. His parents and siblings had been free from strokes, migraines, and dementia.

On admission, the patient's blood pressure was 160/82 mmHg and his pulse rate was 62 beats/min and regular. He was alert and did not have any mood disorder, migraines, scalp alopecia, or spinal osteoarthritis. A neurological examination showed left facial palsy, dysarthria, and dysphagia (pseudobulbar palsy). The Mini-Mental State Examination score was 27 of 30.

Peripheral blood cell counts and biochemical studies showed moderate anemia (hemoglobin, 9.6 g/dL; hematocrit, 28.7%), impaired kidney function (blood urea nitrogen, 69 mg/dL; creatinine, 3.63 mg/dL; urinary protein excretion, 1.04 g/day), dyslipidemia (LDL-cholesterol, 89 mg/dL; HDL-cholesterol, 32 mg/dL; triglycerides, 183 mg/dL), and hyperhomocysteinemia (24.5 nmol/mL). No abnormality was detected in his antinuclear antibody, anticardiolipin antibody, fibrinogen, d-dimer, protein C, protein S, antithrombin III, or antineutrophil cytoplasmic antibody (ANCA) levels.

**Figure 1:**
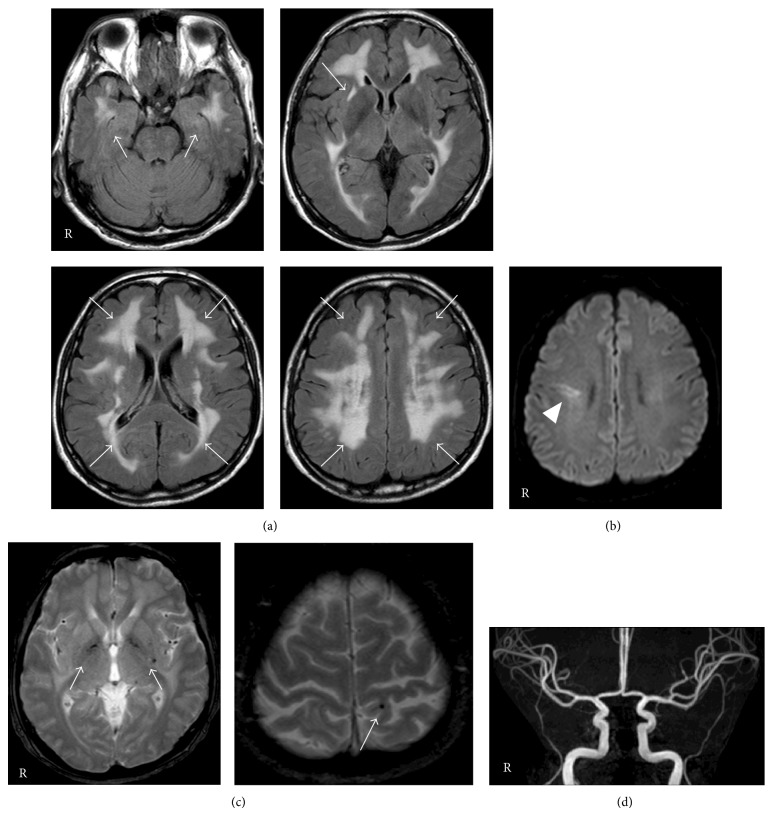
Magnetic resonance images and magnetic resonance angiograms. (a) Fluid attenuated inversion recovery (FLAIR) images demonstrated diffuse hyperintensities in the periventricular and subcortical white matter involving both temporal pole regions and the external capsule (arrow). (b) A hyperintense area on diffusion-weighted images (arrowhead). (c) T2^*^-weighted images showed multiple microbleeds (arrow). (d) Magnetic resonance angiograms.

Cerebral MRI demonstrated diffuse T2 hyperintense areas in the periventricular and subcortical white matter involving bilateral temporal poles and the external capsule (Figure 1). On diffusion-weighted images, a hyperintense area was observed at the right frontal lobe, indicating a recent infarct. T2^*^-weighted images showed multiple microbleeds in the bilateral basal ganglia and subcortical areas. MR angiograms did not show any abnormal findings.

**Figure 2:**
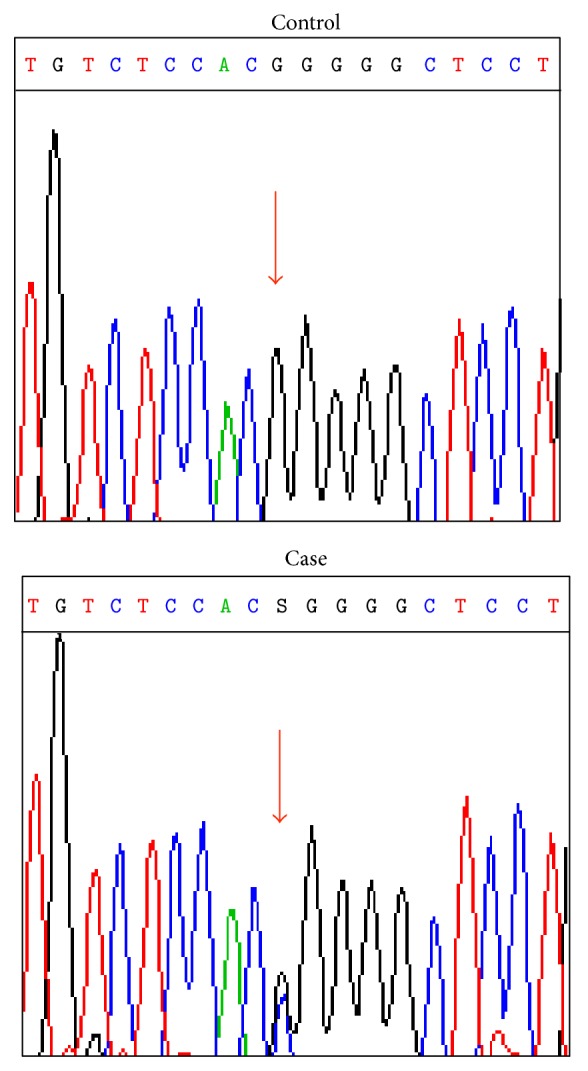
The mutation analysis of the* NOTCH3* gene. The sequence analysis showed a missense mutation, GGG to CGG at the nucleotide 4039 of exon 24 (arrow). The DNA mutation numbering is based on the cDNA sequence (GenBank accession number NM_000435.2), with +1 corresponding to the A of the translational initiation site.

Since he had severe white matter lesions, including the temporal pole, which is characteristic of CADASIL [6], we performed a mutational analysis of the* NOTCH3* gene after he provided informed consent. Genomic DNA was extracted from peripheral blood leukocytes of the patient. Polymerase chain reaction- (PCR-) mediated direct sequencing demonstrated that no mutation was found in exons 3 and 4, which are hot spots in CADASIL. Then, we examined all other exons and splice sites of the* NOTCH3* gene, and G to C transversion was detected in exon 24, c.4039G>C. It changed the codon 1347 from GGG to CGG, resulting in a p.Gly1347Arg substitution (Figure 2). The DNA mutation numbering is based on the cDNA sequence (GenBank accession number NM_000435.2), with +1 corresponding to the A of the translational initiation site. The patient was heterozygous for this missense mutation. We did not find any other mutations or splice variants. The c.4039G>C change was absent in the database of common gene variations in the Japanese population (JSNP), in the Human Gene Mutation Database (HGMD), in the Single Nucleotide Polymorphism database of the NCBI, and in 30 unrelated Japanese controls, who were examined after providing consent according to the ethics code of the Kyushu University Hospital.

The p.Gly1347Arg substitution was present in the EGF-like repeat domain 34 of the Notch3 extracellular domain. According to* in silico* analysis, this substitution was predicted to be “probably damaging” with a score of 0.999 using polymorphism phenotyping-2 (PolyPhen-2, http://genetics.bwh.harvard.edu/pph2/) [7], and it was also predicted to be “deleterious” with a score of −4.930 as determined by Protein Variation Effect Analyzer (PROVEAN, http://provean.jcvi.org/) [8], thus indicating an adverse effect on the structure and function of the protein.

**Figure 3:**
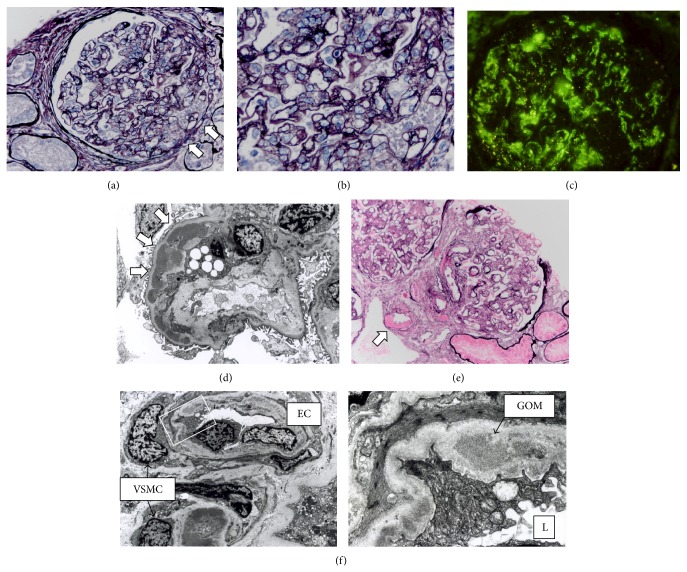
The light microscopic findings and electron microscopic (EM) findings suggestive of MPGN (a–d) or of CADASIL (e, f) in the renal biopsy. (a) A glomerulus showed severe mesangial expansion with mesangiolysis and segmental endocapillary proliferation. Tuft adhesion (arrow) was observed. Periodic acid-silver methenamine (PAM) stain, ×200. (b) Double contours of the glomerular basement membrane with mesangial interposition were also observed. PAM stain, ×400. (c) The glomerulus showed prominent deposition of C3 along the capillary loop. Immunofluorescence stain, ×200. (d) Dilatation of the subendothelial spaces with mesangial interposition was observed. Electron-dense deposits were also observed in subendothelial space (arrow). EM, ×2,500. (e) Severe hyaline changes of the arterioles (arrow) were observed. PAM staining, ×100. (f) Granular deposits were found in the thickened endothelial basement membrane or the basement membrane-like material of arterioles, similar to the findings of GOM. EM, ×2,500 (left panel) and ×12,000 (right panel). Abbreviations: EC: vascular endothelial cell; VSMC: vascular smooth muscle cell; L: vascular lumen; GOM: granular osmiophilic material.

The renal biopsy specimens that had been obtained 28 years earlier showed mesangial cell proliferation with widening of the matrix in almost all glomeruli and an irregular thickened glomerular basement membrane with a “double contour” appearance (Figures 3(a) and 3(b)). In the immunofluorescence studies, the staining of immunoglobulins G, A, M, and C3 was granular and located along the glomerular capillary loop (Figure 3(c)). Electron-dense deposits were confirmed in the subendothelial space by electron microscopy (Figure 3(d)). These findings were consistent with the findings of MPGN. On the other hand, severe hyaline changes of the arterioles were observed, especially from the efferent and afferent arterioles to glomerular arterioles (Figure 3(e)). Granular deposits were also found in many places in the thickened subendothelial basement membrane or the basement membrane-like material of the arterioles, similar to the findings of GOM by electron microscopy (Figure 3(f)). These findings were consistent with CADASIL, whereas the loss of medial vascular smooth muscle cells (VSMCs) was not observed in the patient at that time.

## 3. Discussion

We herein described a case of a CADASIL-like disorder with a novel heterozygous missense mutation of the human* NOTCH3 *gene. The following points are consistent with the diagnostic criteria for CADASIL: (1) multiple subcortical infarcts, (2) diffuse hyperintense lesions, including the external capsule and temporal poles, in T2-weighted images, (3) a heterozygous missense mutation in the EGF-like repeat domain of the* NOTCH3* gene, and (4) granular deposits in the basement membrane of renal arterioles. On the other hand, there were also findings inconsistent with CADASIL: (1) the absence of characteristic symptoms, such as migraines, depression, and cognitive dysfunction, (2) a later onset of symptoms, (3) a sporadic occurrence, (4) some risk factors, including chronic kidney disease, and (5) a noncysteine mutation. Regarding some of the limitations associated with this study, we could not perform another skin biopsy on the patient at this time and also could not perform either MRI, skin biopsy, or a mutational analysis of the* NOTCH3* gene on any of his family members because they did not provide their informed consent.

Many mutations of the* NOTCH3* gene have been detected in CADASIL patients within exons 3 and 4 and are related to a gain or loss of a cysteine residue within the EGF-like repeats [4, 5]. The occurrence of abnormal disulfide bridging and the subsequent protein misfolding would disrupt cellular endocytosis or degradation, change the receptor activation and signal transduction, accumulate GOM, and disrupt the metabolism of smooth muscle cells/pericytes [9, 10]. To our knowledge, the p.Gly1347Arg substitution of the Notch3 protein, which was detected in this case, has not been reported previously. As shown in the prediction analysis, the p.Gly1347Arg substitution located within the EGF-like repeat domain with an additional positive charge may affect the Notch3 signaling. The development of severe white matter lesions is unlikely considering his age and the well-controlled nature of the risk factors. Therefore, the atypical mutation of the* NOTCH3* gene may have led to the impairment of microvascular regulation and consequently led to the development of white matter lesions and the cerebral infarction.

We were able to find a few reports suggesting an association between kidney diseases and CADASIL [11–13]. These cases have some common features in the kidney, including partial to complete VSMCs loss, hyalinosis with thickening of the small artery walls, severe arteriosclerosis, and GOM deposition in the arterioles outside the glomeruli. In the present case, we found severe hyaline changes of arterioles and granular deposits similar to GOM in the basement membrane of renal arterioles, whereas the loss of medial VSMCs was not observed. On the other hand, the decrease in renal function in this case was slow in spite of the presence of histopathological findings like MPGN. The severe hyaline changes in the blood vessels are a common feature of diabetic nephropathy, although the present patient had no history of diabetes mellitus, and granular deposits in the subendothelial basement membrane are not commonly found in diabetic patients. Because podocytes and pericytes, which highly express* NOTCH3* [14], share similar functional and morphological features [15], there is a possibility that the* NOTCH3* gene mutation may affect the function of both the podocytes in the kidney and the pericytes in the brain. Therefore, the* NOTCH3* gene mutation might have contributed to the pathogenesis of the renal arteriopathy in this case.

In summary, we have reported a case of CADASIL-like cerebral lesions and symptoms with a novel heterozygous missense mutation of the* NOTCH3* gene, which also showed chronic renal failure with granular deposits in the renal arterioles. This atypical* NOTCH3* gene mutation might be associated with the impairment not only in the brain, but also in the systemic microvasculature, including that in the kidneys.
